# Hysteroscopic surgery for conservative management in endometrial cancer: a review of the literature

**DOI:** 10.3332/ecancer.2015.505

**Published:** 2015-02-03

**Authors:** Sonsoles Alonso, Teresa Castellanos, Fernando Lapuente, Luis Chiva

**Affiliations:** Department of Gynaecologic Oncology, MD Anderson Cancer Centre, Madrid 28033, Spain

**Keywords:** endometrial cancer, conservative treatment, fertility sparing, hysteroscopy

## Abstract

Endometrial cancer is the most common gynaecologic malignancy, usually diagnosed in postmenopausal women. However, an incidence rate of 2–14% of cases consisting of women under the age of 45 years old has been reported. Multiple reports have described the conservative treatment of this tumour in selected patients with the objective of preserving fertility. In this article, we review the literature to evaluate the results of conservative treatment of endometrial cancer with hysteroscopic resection.

## Introduction

Endometrial cancer is the most common gynaecologic tumour. Its prognosis is generally favourable, with a five-year survival rate of 84.3% for all stages [1]. Federation of Gynaecology and Obstetrics (FIGO) stage is the independent variable that best relates to prognosis. The majority of patients are diagnosed in the initial stages, of which 71–75% of cases with the disease limited to the uterus [2, 3] and with a five-year survival rate of 90% for stage I [3, 4].

Diagnosis was more frequent in postmenopausal women, with an average age of 64–67.3 years old according to studies [1, 5, 6]. However, up to 20% of the cases are diagnosed in the premenopausal stage [7].

According to recent research which studied endometrial cancer in young women and in women of childbearing age, the observed incidence rate was 3.2% for women under 45 [6] and between 2.4% and 5% for women under 40 [3, 7], with an average age of 39.8 years [6] for this group of patients.

Although this incidence rate is not high, diagnosing endometrial cancer in young women who wish to have children presents a challenge for oncological gynaecology. Owing to the delay in maternity, and because of growing evidence that some factors associated with infertility are also associated risk factors for the development of endometrial cancer, the incidence rate of this tumour is found to be even more frequent in patients who are of childbearing age and nulliparous. In fact, up to 54% of premenopausal patients diagnosed were also nulliparous [8].

For this reason, a large number of studies are being carried out to determine the possibility and safety of initiating conservative fertility treatment among this group of patients, and these studies are ever growing.

Endometrial tumours in patients under 45 are often less aggressive, with characteristics suggesting favourable prognosis, since up to 18% of cases are low grade (G1), have not penetrated more than halfway through the myometrium (stage IA) and their histology is endometrioid with positive hormone receptors (type I) [6].

In this way, as a consequence of different studies and research, taking into consideration that although experience is limited, there remains the possibility of initiating a treatment to preserve fertility in patients who have not yet fulfilled their desire to have children. However, in order to bring about said treatment in a way that is oncologically safe, the recommendations are based on compliance with a set of strict selection criteria.

Standard treatment for endometrial cancer begins with staging surgery by means of total hysterectomy with double adnexectomy, peritoneal lavages, and pelvic (and paraaortical depending on the presurgical discovery and risk factors) lymphadenectomy. The approach can be by means of a laparotomy or laparoscopy, preferentially the latter. The possibility of carrying out a selective lymph node biopsy is being developed.

Preservation of fertility treatment includes an initial diagnostic–therapeutic approach by means of a hysteroscopy or dilation and curettage and further study of the sample. A subsequent broad clinical study is then carried out to confirm if the patient fulfills the selection criteria in order to be included in a fertility preservation treatment protocol.

For this, the type of histological grade must be confirmed considering that it will be possible to attempt to preserve fertility in endometrioid histology tumors with low-grade histology (G1, well differentiated).

A subsequent extension study by means of an MRI scan to confirm that the cancer has spread to the myometrium and ovaries is recommended.

Once a patient has been accepted as a candidate for fertility preservation treatment one of the treatment options is recommended, either hormone treatment only, although evidence is very limited, or hysteroscopy combined with hormone treatment according to the experience of the centre.

The most widely discussed treatment in the literature consists of hormone treatment in combination with progestogen in high doses (oral), medroxyprogesterone acetate, and megestrol acetate. Evidence also exists regarding treatment with local progestogens by means of a levonorgestrel intrauterine device (LNG-IUD). This hormone treatment often follows recommended periodical curettages every three months. Other research carried out in combination with this treatment includes local tumour resection by means of a hysteroscopy. However, limited evidence and experience exists for this treatment [9].

This article will review the sets of patients with endometrial cancer submitted for fertility conservation treatment by means of a hysteroscopy for local tumour resection, combined with hormone treatment. We also include a technical surgical analysis, a follow-up, and analysis of remission and pregnancy rates. For this, a systematic indexed search for articles in PubMed between 1975 and June 2014 was carried out.

## Characteristics of the published case studies

Following bibliographical review, we were left with three case studies in which local tumour resection of endometrial carcinoma with hysteroscopy surgery and follow-up treatment with hormone therapy was carried out. We also discovered publications of isolated cases which involved a specific hysteroscopic tumour resection following diagnosis with the aim of preserving the uterus.

The first two studies published are the most significant as regards hysteroscopic surgery treatment because the surgical methodology and technique is well explained, and was written by only those authors that presented the negative arguments of the margins at the time of resection. As the rate of remission is influenced by this confirmation of total tumour resection, this review will analyse the results of these two studies jointly on one hand, and independently of the whole group of case studies on the other hand.

The first cases to be published were from Mazzon *et al* [10] in 2010, with a series of six cases. A more extensive series of 14 cases was published subsequently by Laurelli in 2011 [11]. Therefore, according to the data published by these two authors, a series of 20 cases with a similar surgical technique can be discussed and later analysed in detail, in which complete local tumour resection with negative margins by means of hysteroscopy was carried out.

Additionally, a series published in 2013 by Shan *et al* [12] was studied. This series was based on a prospective observational study in which 14 cases of endometrial cancer and 12 cases of atypical hyperplasia were included. A complete hysteroscopical curettage was carried out in this study and these patients were later treated with hormone therapy. The 14 cases of endometrial cancer included by these authors are encompassed in this review.

Among the case reports, a case was published in 2007 [13] in which a hysteroscopic endomyometrial resection was performed. As with all cases of endometrial cancer observed with a resectoscope, all of the samples were sent with the aim of demonstrating residual illness. All the samples came back negative. In this particular case, part of the myometrium underlying and lateral to the tumour lesion displayed a superficial myometrial invasion.

The approach used is well described and is very similar to that used by Mazzon and Laurelli. It said, this patient was not included in the group of patients reviewed as this patient was 53 years old, and the objective for this conservative treatment to be carried out was only because this patient refused radical treatment (hysterectomy). Six-monthly checks were carried out without evidence of illness or recurrence, five years after surgery.

Finally, in 2014, Marton *et al* [14] published two cases in which endometrial ablation and hysteroscopic resection were done respectively. Although this surgical technique was not described as in the previously selected series, this analysis was included.

There are many series of cases published before the date in which hormone treatment was described as a fundamental therapy in conservative treatment. In many on these cases, diagnosis was made by means of tumour resection or hysteroscopic biopsy, the result being adenocarcinoma. This hysteroscopical procedure assumes tumour resection in many cases, however, the surgical technique was not described in the majority of cases and hysteroscopy was used more as a diagnostic approach than a therapeutic one. These cases were omitted for this reason. We only included those in which hysteroscopy was repeated after diagnosis with the aim of conducting local hysteroscopial surgery.

## Characteristics of the patients selected for conservative surgery

In the two Italian series the age of the patients included is lower than or equal to 40 years old, although the average age of the Mazzon’s group is less. Taking into consideration the total number of patients in these two series, the average age is 35.2 years old with an age range of 26–40 years old (Table 1).

Body mass index (BMI) was evaluated in both groups, however there were no obese patients in the Mazzon’s group, compared to the Laurelli’s group, in which three patients (21%) had a body mass index greater than 30 kg/m^2^.

The patients were nulliparous in the majority of cases: it was an inclusion criterion in the Mazzon’s group, and the Laurelli’s group included three patients who already had one child but still had a strong desire to preserve their fertility (17 out of 20 patients, 85%). Six of the 20 patients (4 in the Mazzon’s group and 2 in the Laurelli’s group) had a past history of infertility, amounting to 30% of the patients (Table 1).

The average age of the 14 patients in the Shan *et al* series [12] was 30.1 years old with an age range of 18–39 years old. The average BMI was 21.8 kg/m^2^ with just one patient having a value greater than 30 kg/m^2^. Every patient in this group was nulligest. Six out of the 14 patients (42.8%) had a history of infertility [12] (Table 1).

The two cases described by Marton *et al* [14] were 30 and 39 years old respectively. One of them was nulligravida and the other was nullipara and secundigravida. Both had a family history of hereditary non-polyposis colorectal cancer (HNPCC). The former was not described in the following series but was considered an exclusion criterion for conservative treatment.

Taking the included series into consideration, we have a total of 36 patients, with an average age of 33.2 years old (18–40), an average BMI of 25.5 kg/m^2^ (7.4–53) and up to 91.6% of them were nulliparous (Table 1).

These patient characteristics, already previously discussed in the literature as histories of nulliparity and sterility are more common in women under 45 with endometrial cancer than in older women with endometrial cancer (61% versus 24%) [1]. These data conform to previous publications studying young patients with endometrial cancer where an average age of 35 years for patients under 40 years, a BMI of 35.1 kg/m^2^, and a history of HNPCC in 3.2% of cases [8].

## Patient selection for conservative surgical treatment

Inclusion criteria for fertility preservation were the same for all groups (Table 2).

Degree of differentiation was used as a first criterion since the tumour should correspond to a low-grade G1 type testing positive for progesterone and estrogen receptors. In all groups hormone receptors were evaluated using immunohistochemistry.

For a better assessment, when the sample originated from another centre, the centre [11] pathologists carried out a review. In the case of Mazzon and Shan a review by two pathologists specialising in gynaecology was carried out independently. In a previous review carried out by our group, we saw that according to the literature, there is a difference between inter and intraobserver of 40% [15], which is why a review by two pathologists is an established recommendation. In the study of two cases by Marton *et al* [14] there is no mention of hormonal receptors or of histological confirmation. There is only an observation that they were dealing with an endometrioid adenocarcinoma.

Therefore, the only candidates for conservative treatment are patients with Grade 1 endometrial adenocarcinomas with an endometrioid histology. This criterion is essential for conservative treatment. Grade 1 tumours are those which are mostly progesterone receptor positive which is fundamental for this treatment [15].

The tumour should be limited to the uterus without myometrial or cervical invasion. The majority of authors demonstrate this using transvaginal ultrasound (TVUS) and magnetic resonance imaging (MRI). Assessing the stage through contrast MRI provides enough precision to rule out myometrial [16] involvement. Precision is increased when used in conjunction with transvaginal ultrasound and when done by expert hands. Both tests are useful for the pre-operative myometrial evaluation [17]. When conservative treatment is used the MRI becomes the most recommended technique for prior assessment [15]. In addition, in the two series carried out by Mazzon and Laurelli, the evaluation of myometrial or cervical involvement is carried out by hysteroscopy.

All patients were evaluated by a general and gynaecological exam, a chest x-ray, and a test for CA 125 serum levels.

Included patients were duly informed of the risks of recurrence or progression of the disease and all signed informed consent forms.

For the assessment of ovarian involvement a routine laparoscopy was carried out on eight patients in the Laurelli’s [11] series after the hysteroscopy was performed. In the Shan’s [12] series a laparoscopy was only carried out on two patients, since it was only indicated for patients for whom ovarian involvement was suspected from the imaging tests.

The incidence of ovarian involvement in patients with apparent Stage 1 disease is 5% according to the literature [18]. However, there are many publications that show a higher incidence of ovarian involvement in groups of young patients. In a recent review by our group, we found an incidence of ovarian involvement in women under 45 with endometrial cancer of between 11% and 29.4% according to the authors [19]. This is a greater incidence than for patients over 45 years of age [6, 20]. Similarly, Evans-Metcalf *et al* showed a significant difference between the two age groups in the univariate analysis. However, when the multivariate analysis was carried out, what actually appeared to be associated with an increase in synchronous ovarian involvement and endometrial cancer was nulliparity [21]. Other authors, like Walsh, in his series with young patients, observed an ovarian involvement of 25% and of these up to 88% of cases involved a synchronic tumour [22]. In any event, be it because of age or nulliparity, we are dealing with a group of patients at risk for ovarian involvement for which it is mandatory to eliminate this possibility, preferably by laparoscopy, as performed in the Laurelli’s group protocol [11].

## Surgical treatment by hysteroscopy

Patients of the two Mazzon’s and Laurelli’s series underwent an initial selection phase during which a diagnostic hysteroscopy was carried out in cases where it had not been performed previously or when there were doubts as to the diagnosis. During which the histology and grade were confirmed by biopsy. For this purpose a 30 degree lens with a diameter of 4 mm was used with a working channel of 5 mm. The hysteroscope was inserted under direct vision without cervical dilatation. The cavity was distended using carbon dioxide (CO2) with an insufflation rate of 35 mL/min, at a pressure of <90 mmHg.

Hysteroscopy allows for a more precise assessment of tumour involvement, as well as adequate biopsy sampling which in turn allows correct assessment of the tumour’s histological grade. This is the most recommended diagnostic technique [1, 15]. In fact, in these two series, 100% of cases the initial diagnostic assessment coincided with that of the definitive one upon surgical resection of the tumour [10, 11].

Laurelli reported that, in the last eight cases, after the diagnostic hysteroscopy was completed, a diagnostic laparoscopy was done to assess the ovaries, and a peritoneal lavage was performed.

Patients who fulfilled the above criteria (Table 2) were subsequently submitted to surgical hysteroscopy.

Mazzon and Laurelli used similar surgical procedures.

Surgical hysteroscopy was performed under general anesthesia. Cervical dilatation to 10 mm was carried out using a Hegar dilator and a 9 mm [10] or 10 mm [11] hysteroscope with 0º lens was inserted. The uterus was distended using a 1.5% glycine solution under gravity inflow of 70 mmHg. The irrigant fluid was collected and monitored carefully. A 5 mm loop with 100 W of cutting power was used for the tumour resection.

Both authors resected the endometrial lesion and a layer of underlying myometrium. In addition, Mazzon described resecting part of the endometrium adjacent to the tumour [10], which he describes as a three-step resection. If the tumour resection resulted positive and the other two (the adjacent endometrium and underlying myometrium) negative they moved forward with conservative treatment, if not standard surgery was carried out.

In addition, Shan *et al* [12] carried out a hysteroscopy curettage during which they resected the major part, if not all of the tumour tissue. This author observed that in eight of the 14 patients (57.15%) with the initial diagnosis of endometrial carcinoma, residual disease was found in the sample after performing the complete endometrial resection with hysteroscopy.

Marton *et al* [14] described in one of their cases, performing an initial polypectomy resulted in the diagnosis of carcinoma. One month later with the intention of surgically resecting the tumour they performed a complete endometrial ablation. In this case Purisol (mannitol/sorbitol mixture) was used to distend the cavity and the intrauterine pressure was limited to 100 mmHg. All samples were negative. In the second case, an initial hysteroscopy was performed where multiple polyps were resected and an adenocarcinoma was diagnosed in one of them. One month later, an endometrial resection was performed again taking multiple endometrium samples that all tested negative.

As we see in these series, surgical hysteroscopy allows for an accurate and safe assessment of the lesion as well as of the rest of the uterine cavity, with a rate of complications of 3% described in the literature [23]. In the series presented no complications were described.

## Hormonal consolidation therapy

Once it was confirmed that a well-differentiated tumour without myometrium invasion and with free resection margins was involved, or that a complete endometrial resection had been done, a hormonal consolidation therapy was begun. For all his patients, Mazzon used megestrol acetate (160mg) daily, beginning the fifth day after the surgery and continuing for six months [10]. Laurelli began a week after the surgery. In six patients, he used the same regimen as Mazzon and in eight patients he used the levonorgestrel intrauterine device (IUD) for 12 month [11]. Shan [12] also used megestrol acetate (160 mg) daily. Unlike other authors, when a partial response was obtained (endometrial cancer regression to simple or complex endometrial hyperplasia with or without atypia) or when a stable disease was involved, Shan increased the dose by 25% for 12 more weeks. If there was partial response at 24 weeks the dose was increased even more (Table 3).

In the cases presented by Marton [14], medroxyprogesterone was used (400 mg) per day for three months in one patient and LNG-IUD for three months in another patient.

The progestagenic agents most widely used for the conservative treatment of endometrial carcinoma are medroxyprogesterone acetate (MPA) and megestrol acetate (MA), followed by the levonorgestrel IUD (LNG IUD). Other agents used include 17-hydroxyprogesterone, norethisterone, oxyprogesterone acetate, hydroxyprogesterone acetate, GnRH analogs, and aromatase inhibitors [24, 25, 26] without any of these having been demonstrated to be superior [27].

## Follow-up

During the first year of follow-up, both Mazzon and Laurelli coincided in performing check-ups every three months. These check-ups consisted of a gynaecological examination, a transvaginal ultrasound, determination of CA-125 serum, and a diagnostic hysteroscopy with biopsies.

A computed tomography (CAT) scan six months after the surgery was also recommended during the follow-up [11].

The follow-up after a year differed a bit between authors. Laurelli continued with quarterly check-ups with the same examinations during the second year, including CAT scans every six months. Thereafter until the fifth year a gynaecological examination, a transvaginal ultrasound, and a CA-125 were performed every six months [11]. However, Mazzon performed check-ups every six months starting from the second year and for another two years [10].

On the other hand, Shan performed monthly check-ups during the treatment, at 12 weeks, with analytics, a CA-125, and transvaginal ultrasound. Subsequently, these same clinical check-ups were carried out every three months with dilatation and curettage every six months.

Complete response was defined as complete absence of tumour cells in the biopsies of the diagnostic hysteroscopies carried out during the follow-up. Shan’s group defined partial response as there was regression from endometrial cancer to typical simple or complex hyperplasia [12]. In Mazzon’s [10] group, patients showing complete response at six months of completing the hormonal treatment could begin attempting pregnancy; in Laurelli’s [11] group, they could begin after 12 months; and in Shan’s [12] after three months.

On the other hand, recurrence was defined as the presence of endometrial cancer in any of the biopsy samples of the follow-up hysteroscopy.

Persistent disease or stable disease was defined as the presence of the same disease that existed prior to treatment. Disease progression was defined as the onset of moderately or poorly differentiated adenocarcinoma.

Patients who showed no response in the initial assessment or in any of the follow-ups were subjected to standard surgery. Shan’s series was an exception: if there was evidence of a partial response or stable disease, the dose was increased for three more months. Complete surgery was recommended in this series when faced with persistent disease or disease progression. Patients who did not achieve pregnancy or who had completed their reproductive desires also underwent complete surgery.

## Patient progress

Patient follow-up was carried out during a minimum of 11 months. If all the series are included this represents a median follow-up time of 40 months, with a range of 13 to 82 months.

In the Laurelli’s series [11], only one relapse was described which occurred five months from the hysteroscopic surgery (1/14, 7%, Table 4). The hormonal consolidation therapy for this patient consisted of the levonorgestrel IUD. The patient underwent complete surgical staging with a final FIGO stage 1A.

If we only take into account the two main series with similar methodology, that is that of Laurelli and Mazzon, we would have a tumour recurrence in 20 patients, which represents a relapse rate of 5%.

However, in the Shan’s [12] series two recurrences were described which represent a rate of 14.2%. These occurred at 10 and 12 months respectively. Both were treated with standard surgery.

Marton *et al* [14] described a recurrence in one of the patients, which was observed when complete surgery was performed after reproductive wishes were completed, 22 months after the initial surgical procedure (Table 4).

With respect to the development of hyperplasia, this was observed in a total of nine patients taking into account all the series (9/36, 25%, Table 4) and in three cases the hyperplasia was atypical. In all cases of hyperplasia without atypia, a resolution was observed with normal check-ups after three months, except in one case which attained a complete response at 9 and 12 months [10]. As a point of interest, in the development of hyperplasia, as well as in the recurrence that occurred in the Laurelli’s series, both patients were obese [11]. In one of the recurrences in Shan’s [12] group the patient had a BMI greater than 30 kg/m^2^.

Therefore, the complete response rate for patients with stage IA G1 endometrial carcinoma treated with fertility preserving surgical hysteroscopy, if we include all the series, is 88.9%, as the tumour recurrence was observed in only four patients (Table 4).

In a previous review of 133 patients conducted by our group, an initial complete response was observed in 75% and an absence of response in 24% of the patients [26]. Of the patients who responded, in up to 66% of cases there was a final complete response. Similarly, in a later revision that included 280 patients with stage IAG1 endometrial adenocarcinoma, a response rate to initial hormonal treatment of 74.6% over an average of six months of follow-up was observed, with persistent disease in 25.4% of the cases [27]. However, in these reviews of hormonal treatments with progestagenic agents as the only conservative treatment, a complete long-term response rate of between 51% [26] and 48.2% [27] is observed. Nevertheless, other authors have reported a higher risk of progression when carrying out follow-ups past 30 months [28] in which case up to 35.4% of patients with an initial complete response may be affected [27], and therefore a close long-term follow-up is recommended.

Therefore the treatment proposed by the authors of surgical resection of the tumour with hormonal consolidation therapy appears to add some benefit given the low relapse rate (11.1%) despite being based on a small series.

## Obstetric results

Patients started attempting pregnancy after completing hormone therapy. Mazzon’s group started at six months [10], Laurelli’s group at 12 months [11] and Shan’s series at three months of confirming complete response [12].

In the Mazzon’s series, all of the patients attempted pregnancy with four of them succeeding, one on two occasions, which represents a rate of pregnancy in this series of 66.6% [10] (Table 5).

In the Laurelli’s group only three patients attempted pregnancy and only one succeeded, so the rate of pregnancy in this series is 33% (1/3) or 7% if we take into consideration all patients in the group (1/14). Similarly, in the Shan’s series, only eight patients in the entire series of 26 attempted pregnancy (including atypical hyperplasia), resulting in two pregnancies, representing a rate of 25% (2/8) or 7.6 % depending on whether you consider all patients or only those that attempted pregnancy (2/26) (Table 5).

Considering that Marton described two clinical cases of pregnancy after conservative treatment for endometrial cancer that were not included in a series of patients these were not added to this analysis.

Pregnancy rates previously described in the literature for exclusively hormonal conservative treatments vary between 34.8–60% [24, 26, 27]. In reality, given that the results were handled quite differently in all the series and that not all of the patients attempted pregnancy, if we only look at Mazzon’s results we get a pregnancy rate of 66% [10], which suggests superiority in achieving pregnancy for the combination of surgery and hormone treatment compared to hormone treatment alone.

There is a commentary published by Park *et al* [29] contemplating the possible adverse effects of hysteroscopic endometrial and myometrial resection prior to hormone therapy for endometrial cancer in cases where preserving fertility was desired. An increase in adhesive syndrome is described after the resection of fibroids with hysteroscopy, as well as the development of fibrosis. According to this author these data could influence the difficulty in achieving a pregnancy after hysteroscopic surgery for endometrial cancer. While it is true that the rate of pregnancy of the Mazzon’s series is quite acceptable and does not appear to be influenced by this hysteroscopic complication, the other series are scarse with regard to the rate of pregnancy and therefore the evidence is still quite limited as far as knowing whether hysteroscopic resection could produce an adhesive syndrome which would prevent a later pregnancy.

Assisted reproduction techniques were used with three patients in Laurelli’s group [11] achieving a pregnancy in one case, while four patients (80%), all of them from Mazzon’s [10] group, became pregnant naturally. In the Shan’s [12] series the two pregnancies were spontaneous, and Marton [14] describes one spontaneous pregnancy and one IVF (in vitro fertilisation) pregnancy. These results differ from the recent reviews that describe a higher percentage of patients who undergo assisted reproduction techniques (66%) [19] and others in which the pregnancy rate is greater when using assisted reproduction techniques than when pregnancy occurs naturally (80% versus 43.2%) [24].

With regard to the time that elapsed before pregnancy is achieved, in Mazzon’s patients this was 24 months on average after completing conservative treatment, with a range of 14–46 [10] months. The patient in Laurelli’s group took 14 months to conceive after completing the treatment.

The obstetric results obtained in the ten births were as follows: all went to term, four were delivered by cesarean and six by normal vaginal delivery. Only Mazzon refers to the weight of the infants, with a median of 3.6 kg and a range of 3.2–4.5 kg [10]. Obstetric results described in the literature vary widely since this information is not always available. The available data show cases of premature birth, and multiple pregnancies, but these results are more related to the use of assisted reproductive techniques than to the history of endometrial carcinoma [24, 25].

## Conclusions

- Fertility preserving treatment is feasible in young patients with stage 1A low-grade progesterone receptor positive endometrioid tumours with no metastatic involvement or risk factors.
- The treatment most widely described is based on progestagenic hormone therapy with close monitoring for long periods.
- Hysteroscopic surgery prior to hormone therapy may improve the rate of recurrence when the resection margins are free, although there is limited evidence. Well-designed, prospective studies should be performed with a well-defined hysteroscopic surgical technique to analyse resection margins and myometrial involvement.
- Evidence regarding the rates of pregnancy for patients in treatment with hysteroscopic surgery is very limited, and may be affected by the endometrial resection itself.

## Figures and Tables

**Table 1.** Clinical characteristics of the patients.

Series	N	Age	Average age	BMI	History if infertility	Nulliparous
Mazzon *et al* [10]	6	< 40	< 33 (27–39)	None were obese	66.6%	6/6 100%
Laurelli *et al* [11]	< 14	< 40	< 38 (26–40)	26 kg/m^2^ (23–53)	< 14	11/14 79%
Total (I)	20		35.2% (26–40)		30%	17/20 85%
Shan *et al* [12]	< 14	< 40	30.1 (18–39)	21.8 kg/m^2^ (7.4–30.5)	42.8%	14/14 100%
Marton *et al* [14]	2	< 40	34.5 (30–39)	-	-	2/2 100%
Total (II)	30	< 40	33.2 (18–40)	25.5 kg/m^2^ (7.4–30.5)		33/36 (91.6%)

(I) Mazzon’s and Laurelli’s series only

(II) All series included

**Table 2.** Assessment prior to conservative treatment.

Criterion	Test
Grade 1	Evaluation by two specialised pathologists
Progesterone receptors +	Immunohistochemistry
Myometrial invasion	TVUS, MRI, hysteroscopic assessment
Ovarian Involvement	MRI, laparoscopy
Advanced Disease	CA 125, Chest x-ray

**Table 3.** Hormonal consolidation therapy.

Hormonal therapy	Cases	Starting point after surgery	Duration
MA (160mg/d)	26	Day 5 or 7	6 months
Medroxyprogesterone acetate (400 mg/day)	1	-	3 months
LNG-IUD	1	-	3 months
LNG-IUD	8	Day 7	12 months

**Table 4.** Response rates: relapse and result.

	Median follow-up (months)	Recurrence	Persistent disease	Time to recurrence	Hyperplasia without atypia	Atypical hyperplasia
Laurelli [11]	40 (range 13–79)	1/14 (7%)	-	5 months	1/14 (7%)	0
Mazzon [10]	50.5 (range 21–82)	0%	-	-	3/6 (50%)	1/6 (16.6%)
Shan [12]	34.7 (range 15–66)	2/14 (14.2%)	3/14 (21%)	10 and 12 months	2/14 (14%)	1/14 (7.1%)
Marton [14]	11 and 22 months	½ 50%		22 months	-	½ 50%
Total	40 (range 11–82)	4/36 (11.1%)		12.2 months	6/36 (16.6%)	3/36 (8.3%)

**Table 5.** Obstetric results.

Series	N	Method	Duration	Number of pregnancies	Obstetric outcome	Rate of pregnancy
Mazzon	Four	Natural	24 months (range 14–46)	Five	Four cesarean section One vaginal delivery	66.6%
Laurelli	One	ART	14 months	One	One vaginal delivery	33.3%
Shan	Two	Natural	-	Two	One vaginal birth, another unknown	25%
Marton	Two	Natural and ART	3 and 10 months	Two	Two vaginal delivery	100%
Total	9		Range 3–46 months	Ten		

ART Assisted Reproductive Technology
